# Louqin Zhisou Decoction Inhibits Mucus Hypersecretion for Acute Exacerbation of Chronic Obstructive Pulmonary Disease Rats by Suppressing EGFR-PI3K-AKT Signaling Pathway and Restoring Th17/Treg Balance

**DOI:** 10.1155/2019/6471815

**Published:** 2019-01-20

**Authors:** Feng Feng, Jianchao Du, Yufeng Meng, Fang Guo, Cuiling Feng

**Affiliations:** ^1^Beijing University of Chinese Medicine, Beijing 100029, China; ^2^Dongzhimen Hospital, Beijing University of Chinese Medicine, Beijing 100700, China; ^3^Peking University People's Hospital, Beijing 100044, China; ^4^Beijing Hospital of Traditional Chinese Medicine Shunyi Branch, Beijing 101300, China

## Abstract

Airway mucus hypersecretion is the main pathogenic factor in acute exacerbation of chronic obstructive pulmonary disease (AECOPD) and the control of mucus secretion is closely associated with survival. Louqin Zhisou decoction (LQZS) has been found to improve lung function and reduce sputum in AECOPD patients, but the mechanism remains unclear. This study aimed to explore the mechanism of LQZS against mucus hypersecretion in lung tissues of rat AECOPD model. Wistar rats were used to establish AECOPD model by intratracheal instillation of LPS in combination with the continuous cigarette smoking. Rats were administrated LQZS/clarithromycin (CAM)/distilled water via gavage every day and all rats were sacrificed after 30 days. BALF and lung tissues were obtained. Lung morphology, cytokines levels, MUC5AC mRNA transcription and protein expression, phosphorylation of the EGFR-PI3K-AKT signaling pathway, and molecules involved in Th17/Treg balance were evaluated. The results demonstrated that LQZS protected rats from decline in pulmonary function and ameliorated lung injury. LQZS treatment decreased the number of goblet cells in airway and suppressed MUC5AC mRNA and protein expression of lung tissues. Furthermore, LQZS attenuated the level of phospho-EGFR, phospho-PI3K and phospho-AKT in AECOPD rats. In addition, LQZS could inhibit the production of proinflammatory cytokines in BALF, including IL-6 and IL-17A and downregulate the secretion of NE and MCP-1, indicating that LQZS could limit inflammatory responses in AECOPD. Moreover, LQZS reversed ROR*γ*t and Foxp3 expression, the key transcription factors of Th17 and Treg, respectively. In conclusion, this research demonstrated the inhibitory effects of LQZS against mucus hypersecretion in AECOPD via suppressing EGFR-PI3K-AKT signaling pathway and restoring Th17/Treg balance.

## 1. Introduction

Chronic obstructive pulmonary disease (COPD) is one of the most common health problems around world with symptoms such as cough, sputum, and dyspnea [1]. It is characterized by persistent airflow limitation which always aggravated by airway inflammation. Acute exacerbation of chronic obstructive pulmonary disease (AECOPD) is an important event in the progression of COPD because it negatively impacts the lung function, rates of hospitalization, and survival period [1]. The main pathological features of AECOPD are obstructive bronchiolitis, emphysema, and mucus hypersecretion [2]. In the normal airways, the mucus layer coating on the epithelium is thin, whereas in mucosa of AECOPD patients, excess mucus secretion could be found [3, 4]. Since redundant mucus can narrow bronchial airways and facilitate bacterial colonization, mucus hypersecretion contributes to lung function decline and bacterial infections in AECOPD [5]. Mucus hypersecretion is not only pathology products but also risk factors for COPD aggravation.

The major components of mucus are mucins [6]. These complex glycoproteins are mainly synthesized under the regulation of mucin (MUC) gene. Mucin5AC (MUC5AC) is the primary gene expressed by airway epithelial goblet cells [7]. Stimuli such as cigarette smoke [8], bacterial products [9], cytokines (e.g., IL-13 [10]), chemokines (e.g., CXCL-8 [11] or MCP-1 [12]), and neutrophil proteases (e.g., neutrophil elastase [13, 14]) upregulate MUC5AC productions and induce goblet cells hyperplasia and/or metaplasia. It has been reported that MUC5AC gene expression is stimulated by numerous signaling pathways, especially epidermal growth factor receptor (EGFR) [15]. Binding of EGFR to its ligand leads to phosphorylation of EGFR that activates downstream signaling cascades including extracellular-signal-regulated kinases1/2 (ERK1/2), phosphatidylinositol-4,5-bisphosphate 3-kinase (PI3K)/AKT, and p38 mitogen-activated protein kinase (MAPK) transduction pathway. These signals are transferred into nuclear to enhance MUC5AC synthesis by transcription factors (e.g., AP-1 and nuclear factor-*κ*B (NF-*κ*B)). The transcription factor FOXA2 has inhibitory effect in mucin production and goblet cell differentiation mediated by EGFR [16] and FOXA2 expression was decreased via EGFR activation [17]. After mucin proteins have synthesized, they are tightly packed into intracytoplasmic granules of airway goblet cells. Mucin granules remain stable in the normal condition thus mucins secrete at low rate until triggered by extracellular stimuli especially neutrophil elastase (NE). NE expressed by neutrophils is essential for mucins production, exocytosis and degradation [18].

Imbalance between T helper (Th) 17 cells and regulatory T (Treg) cells plays a potent role in neutrophils recruitment and activation of airway epithelium. Th17 cells, CD4+ T lymphocytes, promote inflammation of airway by producing cytokine IL-17A which induces neutrophil chemokine secretion [19]. It was reported that IL-17A expression increased in the bronchial mucosa of COPD patients [20]. On the contrary, CD4+ CD25+ Foxp3+ Treg cells with immunoregulatory functions suppress immune response by releasing inhibitory cytokines such as IL-10 and TGF-*β*. In COPD patients, Treg cells from peripheral blood decreased compared with healthy subjects [21]. Therefore, Th17/Treg imbalance is vital in the development of airway inflammation and mucus hypersecretion in COPD.

Several medications (e.g., macrolides, corticosteroids, N-acetylcysteine, carbocysteine, and muscarinic receptor antagonists) were found efficient in reducing mucus hypersecretion in experimental models, but there is no evidence of beneficial effects in clinical [10]. Among these therapies, macrolides (including azithromycin, clarithromycin, erythromycin, roxithromycin, and telithromycin) can both cause antibacterial effects and decrease inflammatory response [22]. In recent years, Traditional Chinese Medicine (TCM) has shown benefits in COPD treatments. According to TCM theory, “phlegm-heat obstructing lung” is one of the main syndrome types of AECOPD. Louqin Zhisou (LQZS) decoction, a Chinese herbal formula, is widely used in clinical of Dongzhimen Hospital in China. It contains eight herbs: Gualoupi (Pericarpium Trichosanthis), Huangqin (Radix Scutellariae), Lianqiao (Fructus Forsythiae), Loulu (Radix Rhapontici), Zhebeimu (Bulbus Fritillariae Thunbergii), Qingbanxia (Rhizoma Pinelliae Preparata), Qianhu (Radix Peucedani), and Jiegeng (Radix Platycodonis). Our previous study has proved that LQZS decoction can alleviate clinical symptoms and expectoration in AECOPD patients [23]. However, the mechanism has not been determined. In an early study, we successfully established rat AECOPD model by intratracheal instillation of LPS in combination with cigarette smoke [24]. This study was designed to investigate the mechanism of LQZS effect on mucus hypersecretion by regulating EGFR-PI3K-Akt signaling and Th17/Treg balance in AECOPD rats.

## 2. Materials and Methods

### 2.1. Medicine Preparation

Clarithromycin (CAM) purchased from Livzon Pharmaceutical Group Co., Ltd. (Zhuhai, China), was diluted to the concentration 5mg/mL by distilled water. LQZS is composed of 15g Gualoupi (Pericarpium Trichosanthis), 10g Huangqin (Radix Scutellariae), 10g Lianqiao (Fructus Forsythiae), 10g Loulu (Radix Rhapontici), 10g Zhebeimu (Bulbus Fritillariae Thunbergii), 10g Qingbanxia (Rhizoma Pinelliae Preparata), 10g Qianhu (Radix Peucedani), and 10g Jiegeng (Radix Platycodonis). All herbs were prepared into granules by Beijing Kangrentang Pharmaceutical Co., Ltd. (Beijing, China). The granules were suspended with distilled water and brought to a final concentration of 0.84g/mL.

### 2.2. Animals Model Establishment and Treatment

Forty-eight Wistar rats (male, six-week-old, 180-220g) were purchased from Vital River Laboratory Animal Technology Co., Ltd. (Beijing, China) and randomly divided into four groups (n = 12 in each group): Control group, which was normal rats undergoing no treatment; AECOPD group, which was AECOPD rats without treatment; CAM group, which was AECOPD rats treated with CAM; and LQZS group, which was AECOPD rats given LQZS.

All rats were maintained on a 12h dark/light cycle in a temperature-controlled room (25°C) and had sufficient food (sterile rat chow) and water (sterile). After 7 days' accommodating to the facility, the rats were under model establishment and treatment. The experimental protocols were approved by the Experimental Animal Ethics Committees of Peking University People's Hospital.

Rat AECOPD model was established by using cigarette smoke combined with intratracheal instilling lipopolysaccharide (LPS, Sigma, USA). Specifically, on day 1, day 14, and day 28, each rat was instilled 200*μ*g LPS (prepared as 1mg/mL solution in saline) through the throat of rats using a 18G remaining catheter after anesthesia with 5% isoflurane. Then the rats were swayed softly on a board for 20 seconds to ensure the uniform distribution of LPS in the lungs. On each day of days 2-13, days 15-27, and days 29-30, the rats were kept in a covered box (70 cm × 60 cm × 60 cm) and exposed to the smoke from eight continuously burned cigarettes for 30 minutes. Commercial cigarettes (Daqianmen Filter Cigarette, Shanghai, China) were provided by Shanghai Tobacco Industry Co., Ltd., and each cigarette contained 0.9 mg nicotine, 14 mg CO, and 12 mg tar oil, according to the manufacturer's specifications. The successful AECOPD model was estimated by lung function and pulmonary pathology.

Rats in Control and AECOPD groups were intragastrically given distilled water (10mL/kg·bw); CAM group was administrated CAM solution (10mL/kg·bw) by gavage; LQZS group was given LQZS solution (10mL/kg·bw). All treatments were initiated day 1, q.d., for 30 days. On day 31, all rats were anesthetized by intraperitoneal injection of pentobarbital sodium (50mg/kg) and determined pulmonary functions before sacrificed by blood collection from abdominal aorta.

### 2.3. Pulmonary Function Examination

After the rat was anesthetized, a plastic cannula was placed in the trachea via a small transverse incision made in the neck. Then the cannula was connected to an animal pulmonary functionality test machine AniRes2005 (Bestlab, Beijing, China) to measure the maximal voluntary ventilation (MVV), forced vital capacity (FVC), forced expiratory volume in 0.3 second (FEV0.3), and FEV0.3/FVC%.

### 2.4. HE and AB/PAS-Staining

After about 8mL blood sample was collected, opened the rat's chest, tightened the hilum of right lung, and isolated the middle lobe. The lung tissue was fixed by 4% paraformaldehyde for 48h, dehydrated in alcohol for 5h, embedded in paraffin, cut into 4*μ*m sections, and stained with hematoxylin and eosin (H&E) to evaluate general morphology under Leica light microscope. Alcian Blue/Periodic Acid-Schiff (AB/PAS) staining was applied to detect goblet cell metaplasia of bronchial epithelium. The images of lung tissues were captured by microscope. AB/PAS-positive area and total area of corresponding bronchial epithelium were measured by the software Image-Pro Plus 6.0. Data were presented as the ratio of AB/PAS-positive area to the total area.

### 2.5. Immunohistochemistry (IHC)

Tissues paraffin was sliced into 3*μ*m continuous sections. The sections were heated in 1mM ethylenediaminetetraacetic acid (EDTA) at 95°C for 20 minutes, incubated with 3% hydrogen peroxide for 20 minutes, and blocked by 10% goat serum for 90min at room temperature. Section was then incubated overnight at 4°C with 100*μ*L of mice monoclonal IgG anti-rat MUC5AC antibody (abcam, USA, dilution 1:200), 100*μ*L of rabbit polyclonal IgG anti-rat ROR*γ*t antibody (abcam, USA, dilution 1:50), 100*μ*L of mice monoclonal IgG anti-rat Foxp3 antibody (abcam, USA, dilution 1:100), 100*μ*L of rabbit polyclonal IgG anti-rat NE antibody (abcam, USA, dilution 1:2000), and mice monoclonal IgG anti-rat MCP-1 antibody (proteintech, China, dilution 1:200). The sections were then rinsed in 1× PBS and incubated with HRP-conjugated goat anti-rabbit second antibody (zsbio, China, dilution 1:400) or goat anti-mice second antibody (zsbio, China, dilution 1:400) for 1h at room temperature. After being rinsed in 1×PBS, the sections were developed with 3,3-diaminobenzidine tetrahydrochloride (DAB). The images were captured by Leica light microscope. The brown stains were positive for target proteins.

### 2.6. ELISA

5mL of bronchoalveolar lavage fluid was collected from the left lung and centrifuged at 3000 rpm for 10min at 4°C. The supernatant was saved for measurement of inflammation cytokines IL-6, TNF-*α*, TGF-*β*, IL-10, and IL-17A by using ELISA kits (MultiScience, Hangzhou, China) according to the manufacturer's instructions. The absorbance was measured at a wavelength of 450nm with a microplate reader (Bio-Rad).

### 2.7. Quantitative Real-Time PCR (qPCR)

Total RNA was extracted from posterior lobe of the right lung by using Trizol-Reagent (Invitrogen, USA) following the manufacturer's instructions. Reverse transcription (RT) reaction was proceeded by using Access RT-PCR system (Promega, USA), and real-time PCR reactions were performed by using a SYBR® Green Super Mix kit (Bio-Rad, USA). The reaction conditions included 95°C for 10 min to activate, followed by 40 cycles of 95°C for 10 s, 60°C for 30 s, and 72°C for 1 min. In each run, melting curves were generated to evaluate specific amplification of target genes. The primers of MUC5AC, ROR*γ*t, Foxp3, IL-17, IL-10, NE, MCP-1, and GAPDH served as internal control were designed and synthesized by Sangon Biotech Co., Ltd. (Shanghai, China) and sequences were shown in Table 1. 2^−ΔΔCT^ was used to calculate the relative expression of the genes.

### 2.8. Western Blot Analysis

20mg lung tissues were homogenized with 200*μ*l RIPA buffer containing protease inhibitors and centrifuged at 4°C, 14000g for 5min to extract total proteins. The supernatant was saved at -80°C and the protein concentration was determined by Bradford assay (BSA) method. 50*μ*g proteins were mixed with 4×SDS loading buffer, denatured in a water bath for 5min at 95°C, separated by 8% SDS-PAGE, and transferred to PVDF membranes. The membranes were blocked with 5% bovine serum albumin (BSA) in 1×TBS at room temperature for 2h. Then the filters were incubated with primary antibodies, mice monoclonal IgG anti-rat MUC5AC antibody (abcam, USA, dilution 1:1000), rabbit polyclonal IgG anti-rat EGFR antibody (affinity, China, dilution 1:1000), rabbit polyclonal IgG anti-rat phospho-EGFR antibody (affinity, China, dilution 1:1000), rabbit polyclonal IgG anti-rat PI3K antibody (affinity, China, dilution 1:1000), rabbit polyclonal IgG anti-rat phospho-PI3K antibody (affinity, China, dilution 1:1000), rabbit polyclonal IgG anti-rat AKT antibody (affinity, China, dilution 1:1000), rabbit polyclonal IgG anti-rat phospho-AKT antibody (affinity, China, dilution 1:1000), rabbit polyclonal IgG anti-rat ROR*γ*t antibody (abcam, USA, dilution 1:500), mice monoclonal IgG anti-rat Foxp3 antibody (abcam, USA, dilution 1:1000), rabbit polyclonal IgG anti-rat NE antibody (abcam, USA, dilution 1:500), mice monoclonal IgG anti-rat MCP-1 antibody (proteintech, China, dilution 1:1000), and rabbit polyclonal IgG anti-rat *β*-actin (proteintech, China, dilution 1:2000) served as internal control at 4°C overnight. After being washed with 1×TBST, the membranes were incubated with horseradish peroxidase- (HRP-) conjugated second antibody at room temperature for 1h. At last, the bands were scanned by gel-imaging system with Enhanced Chemiluminescence (ECL) reagent (Beyotime, China) and measured by Image J software (NIH, MD, USA).

### 2.9. Statistical Analysis

SPSS 22.0 software (IBM; Armonk, NY, USA) was used for data analysis. The result was expressed as mean ± SD. The statistical differences between groups were determined by One-way analysis of variance (ANOVA).* P* < 0.05 was considered significant.

## 3. Results

### 3.1. LQZS Improved the Pulmonary Function in Rat AECOPD Model

Compared to Control group, there were evidently declines in lung function parameters such as FEV0.3, FEV0.3/FVC% and MVV (*P* < 0.01) which reflected development of emphysema and airway enlargements in AECOPD group. The results of treatment groups showed that CAM and LQZS could improve MVV, and the effect on MVV of LQZS group is more significantly than CAM group (*P* < 0.05) (Table 2).

### 3.2. LQZS Reduced Pulmonary Histopathological Injury in Rat AECOPD Model

**Figure 1:**
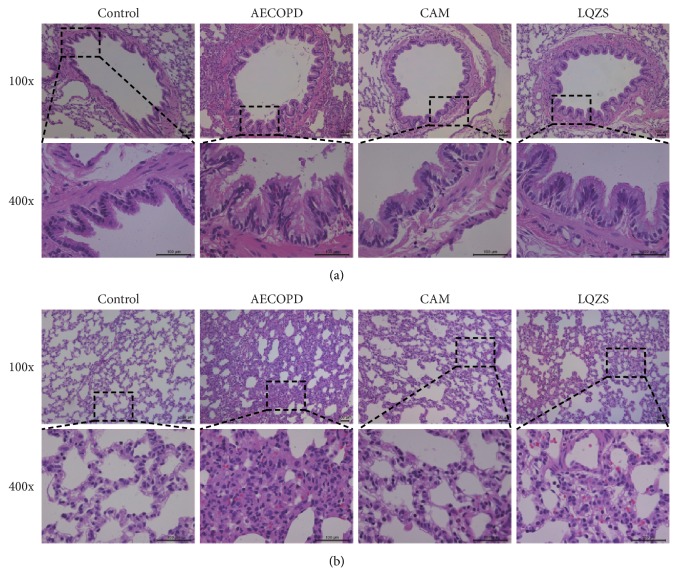
Lung morphology of each group. (a) Pathological changes in the airway structure (H&E staining magnification ×100 and ×400). Control group showing a normal structure of airway; AECOPD group exhibiting noticeable changes, with thickened airway, loss of cilia, submucosal gland hyperplasia, and hypertrophy; CAM group and LQZS group showing less airway inflammation. (b) Pathological changes in the alveoli (H&E staining magnification ×100). Control group demonstrating regular morphology of alveoli; AECOPD group showing inflammatory cell infiltration, broken alveolar walls, widen alveolar septum, and hyperemia; CAM group and LQZS group showing lower alveolar destruction.

As shown in Figures 1(a) and 1(b), in Control group the structure of bronchial epithelium was intact with little inflammatory cell infiltration and submucosal gland hyperplasia. The alveolar walls and capillary maintained their integrity without bleeding and edema. Compared with Control group, there were obvious histological injuries in AECOPD group such as thickened airway, loss of cilia, submucosal gland hyperplasia and hypertrophy, inflammatory cell infiltration, broken alveolar walls, widen alveolar septum and capillary congestion. In both CAM group and LQZS group, pulmonary injuries and inflammatory infiltration could be observed but were significantly attenuated compared with AECOPD group.

### 3.3. LQZS Decreased Goblet Cell Hyperplasia in Rat AECOPD Model

**Figure 2:**
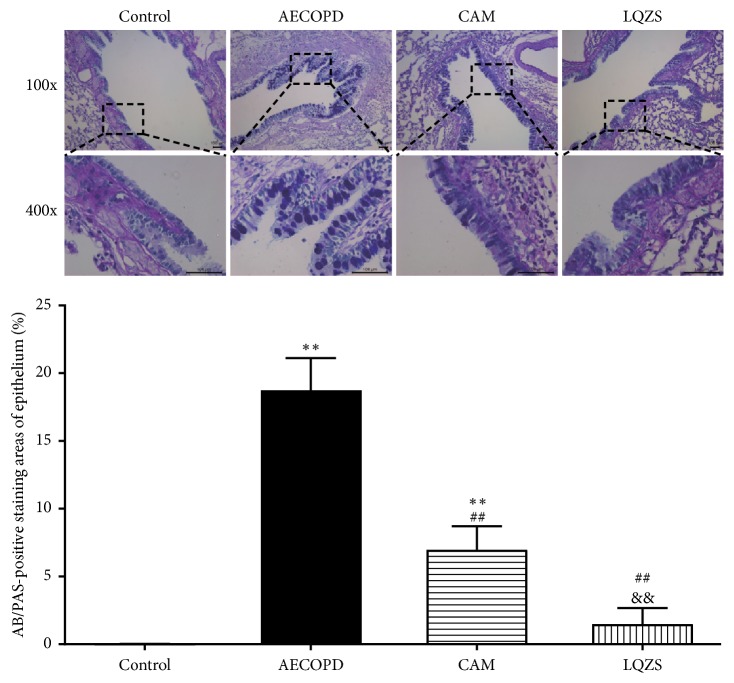
Effect of LQZS on goblet cell hyperplasia in the bronchial epithelium of rats with AECOPD. Goblet cell of bronchial epithelium detected by AB/PAS-staining (magnification ×400). The positive staining was presented blue or purple. Quantification of goblet cell hyperplasia in the airway (n=6). AB/PAS-positive rates were determined by the ratio of AB/PAS-positive area to the total bronchiolar epithelial area. The values were expressed as mean ± SD. One-way ANOVA was adopted for statistical analysis. ^*∗∗*^Compared to Control group* P* < 0.01; ^##^compared to AECOPD group* P* < 0.01; ^&&^compared to CAM group* P* < 0.01.

Goblet cells in the airway epithelium could be observed under the light microscopy by the use of AB/PAS-staining. The positive staining area rates of airway epithelium were 0.02 ± 0.02%, 18.73 ± 2.38%, 6.98 ± 1.74% and 1.49 ± 1.18% in Control group, AECOPD group, CAM group, and LQZS group, respectively. There was a significantly increase of positive stains indicating goblet cell hyperplasia in AECOPD group compared with Control group (*P* < 0.01). It could be figured that both CAM and LQZS attenuated goblet cell hyperplasia of bronchial epithelium (*P* < 0.01), while the effect of LQZS was more potent (*P* < 0.01) (Figure 2).

### 3.4. LQZS Attenuated Mucus Hypersecretion in Rat AECOPD Model via EGFR-PI3K-AKT Signaling Pathway

**Figure 3:**
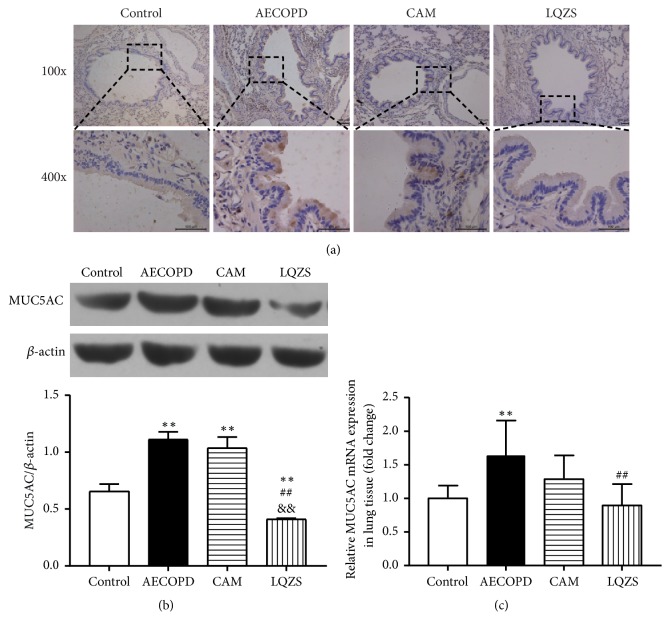
Effect of LQZS on MUC5AC synthesis and expression in lung tissues of rats with AECOPD. (a) Immunohistochemical staining of MUC5AC in bronchial epithelium (magnification ×100 and ×400). (b) Estimate of MUC5AC expressions through Western blot. *β*-actin was used as an internal control. The values were shown as proportions of MUC5AC to *β*-actin optical density. (c) Changes in relative mRNA levels of MUC5AC. The values were expressed as mean ± SD. One-way ANOVA was adopted for statistical analysis. ^*∗∗*^Compared to Control group* P* < 0.01; ^##^compared to AECOPD group* P* < 0.01; ^&&^compared to CAM group* P* < 0.01.

IHC, qPCR, and Western blot were performed to evaluate the effects of LQZS on MUC5AC synthesis and expression. As shown in Figure 3(a), positive stains were hardly detected in normal airway, whereas AECOPD group resulted in significant brown stains in bronchial epithelium. Compared with AECOPD group, positive products were reduced with CAM and LQZS treatment. Compared with CAM group, the inhibitory effect on MUC5AC expression in LQZS group was more obvious for few positive stains observed. Meanwhile, Western blot analysis on MUC5AC protein expression in lung tissues showed that cigarette smoke and intratracheal LPS led to high MUC5AC expression which was markedly reduced by LQZS treatment (Figure 3(b)). As for qPCR results, expression of MUC5AC mRNA was noticeably upregulated compared with Control group, while in LQZS group the transcription was largely attenuated (Figure 3(c)).

**Figure 4:**
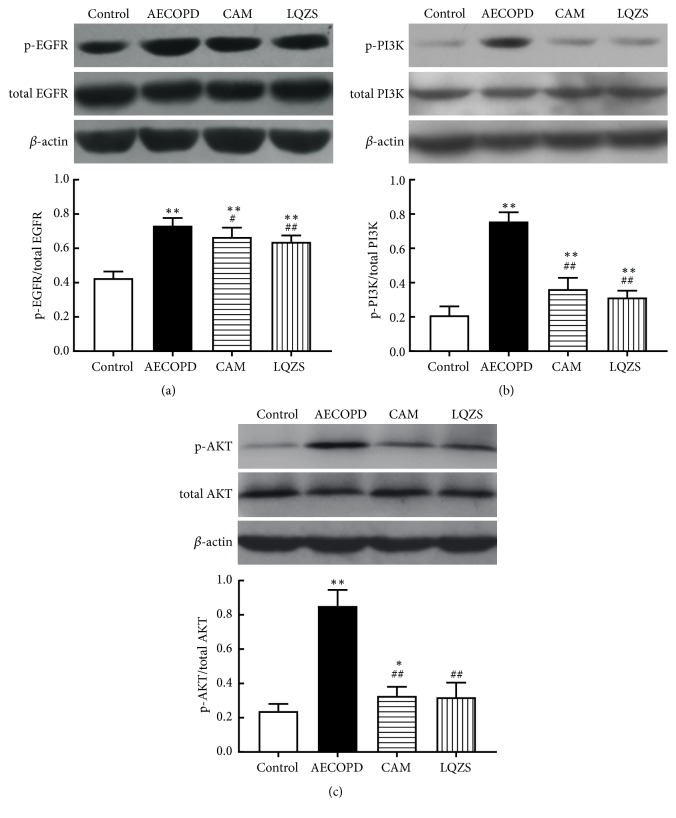
Effect of LQZS on phosphorylation levels of EGFR, PI3K, and AKT in lung tissues of rats with AECOPD. (a) Quantitative detection of ratios of phospho-EGFR to total EGFR. (b) Quantitative detection of ratios of phospho-PI3K to total PI3K. (c) Quantitative detection of ratios of phospho-AKT to total AKT. The values were expressed as mean ± SD. One-way ANOVA was adopted for statistical analysis. ^*∗*^Compared to Control group* P* < 0.05, ^*∗∗*^*P* < 0.01; ^#^compared to AECOPD group* P* < 0.05, ^##^*P* < 0.01.

To further investigate the mechanisms of LQZS on MUC5AC downregulation, we performed Western blot analysis to detect phosphorylation of EGFR, PI3K, and AKT proteins in rat lungs. Results showed that there was a significant increase in phospho-EGFR, phospho-PI3K, and phospho-AKT of AECOPD group compared with Control group. The phosphorylation was decreased to varying degrees in both CAM group and LQZS group with statistical significance (Figure 4).

### 3.5. LQZS Suppressed Inflammation in Rat AECOPD Model via Restoring Th17/Treg Balance

**Figure 5:**
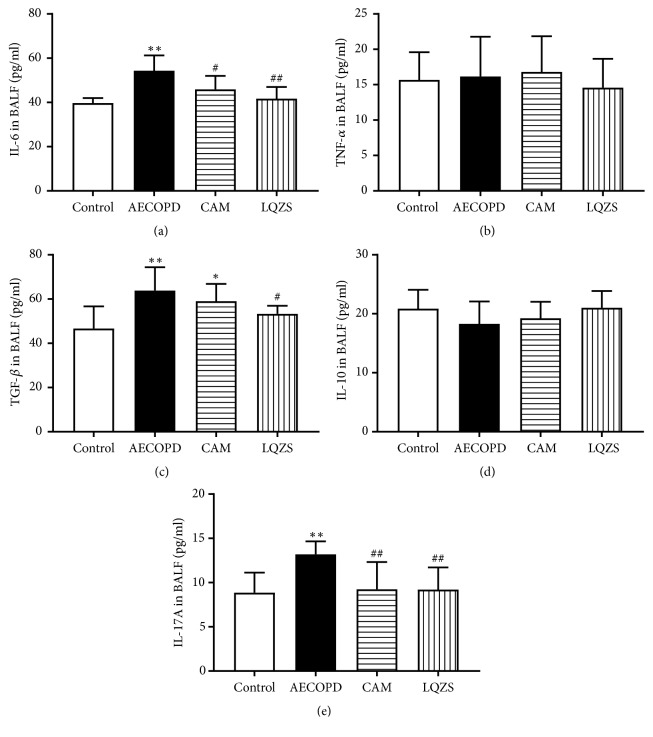
Effect of LQZS on cytokines IL-6 (a), TNF-*α* (b), TGF-*β* (c), IL-10 (d), and IL-17A (e) and in BALF of rats with AECOPD. The values were expressed as mean ± SD. One-way ANOVA was adopted for statistical analysis. ^*∗*^Compared to Control group* P* < 0.05, ^*∗∗*^*P* < 0.01; ^#^compared to AECOPD group* P* < 0.05, ^##^*P* < 0.01.

To detect the effect of LQZS on pulmonary inflammation and Th17/Treg balance markers, we measured the cytokines including IL-6, TNF-*α*, TGF-*β*, IL-10, and IL-17A in BALF by ELISA. ELISA results (Figure 5) showed that the levels of IL-6, TGF-*β*, and IL-17A in AECOPD group were upregulated compared to Control group (*P* < 0.01). Compared with those in AECOPD group, the levels of IL-6, IL-17A (*P* < 0.01), and TGF-*β* (*P *< 0.05) in LQZS groups and IL-6 (*P* < 0.05) and IL-17A (*P* < 0.01) in CAM were significantly decreased. There was no statistical significance comparing the level of IL-10 and TNF-*α* in 4 groups (*P* > 0.05).

**Figure 6:**
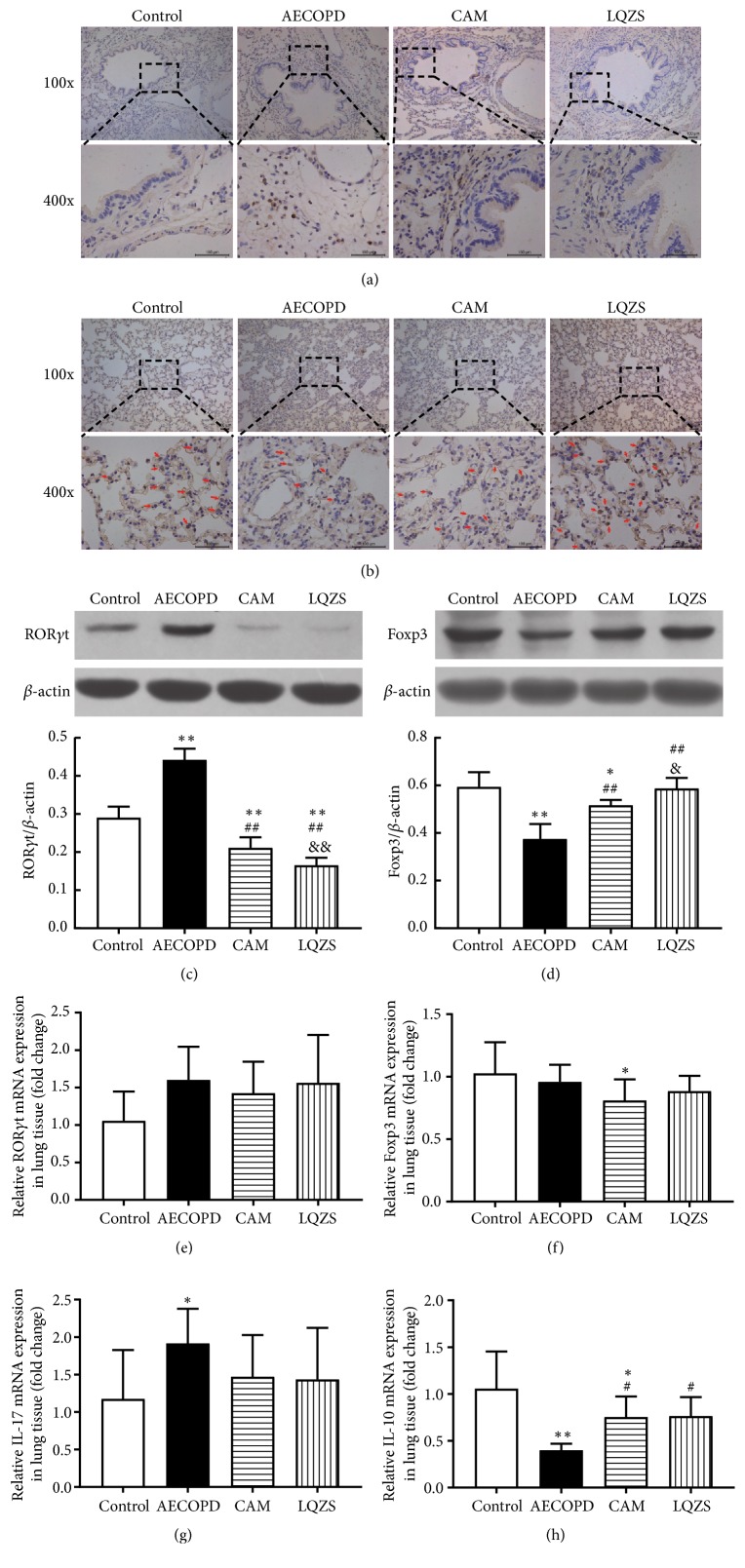
Effect of LQZS on repairing Th17/Treg imbalance in lung tissues of rats with AECOPD. Immunohistochemical staining of ROR*γ*t (a) and Foxp3 (b) proteins in lung tissues (magnification ×100 and ×400). Estimate of ROR*γ*t (c) and Foxp3 (d) expressions through Western blot. *β*-actin was used as an internal control. The values were shown as proportions of target proteins to *β*-actin optical density. Changes in relative mRNA levels of ROR*γ*t (e), Foxp3 (f), IL-17(g), IL-10 (h) in lung tissues. The values were expressed as mean ± SD. One-way ANOVA was adopted for statistical analysis. ^*∗*^Compared to Control group* P* < 0.05, ^*∗∗*^*P* < 0.01; ^#^compared to AECOPD group* P* < 0.05, ^##^*P* < 0.01; ^&^compared to CAM group* P* < 0.05, ^&&^*P* < 0.01.

To further investigate the anti-inflammation effect of LQZS via regulating Th17/Treg balance, we used IHC, qPCR, and Western blot to detect Th17/Treg-related transcription factors markers. As shown in Figure 6(a), few positive stains which reflected the level of ROR*γ*t protein were found in normal lung tissues. In contrast to Control group, tissue surrounding airways and vessels from AECOPD group reflected a large number of positive products. Compared with AECOPD group, ROR*γ*t protein expression was reduced with CAM and LQZS treatment, especially in LQZS group. However, in terms of Foxp3, cells with stained nuclear in alveolar wall and interstitial were decreased under the influence of cigarette and LPS. The positive cells with darker colour nuclear were rising in CAM and LQZS groups to varying degrees (Figure 6(b)). Bands determined by Western blot analysis showed the same trends of ROR*γ*t and Foxp3 proteins with statistical significance (Figures 6(c) and 6(d)). Furthermore, IL-10 mRNA, almost exclusively transcribed by Treg cells, was found upregulated by CAM and LQZS compared with the suppression in AECOPD group (Figure 6(e)).

**Figure 7:**
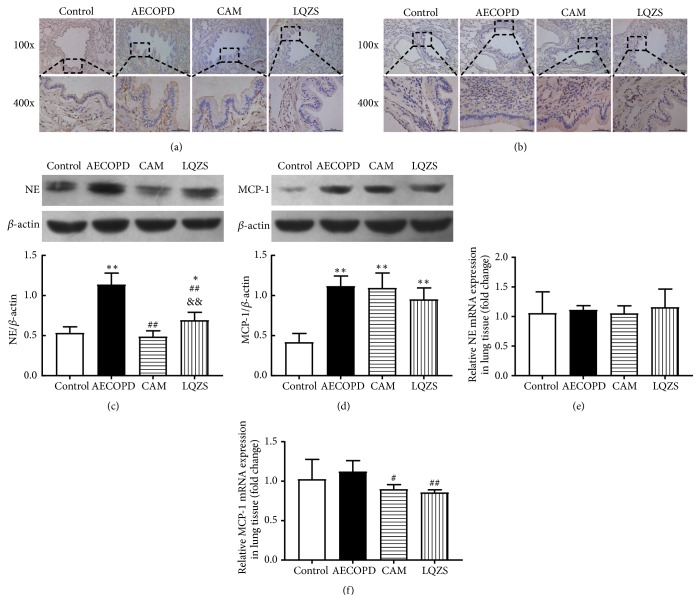
Effect of LQZS on NE and MCP-1 synthesis and expressions in lung tissues of rats with AECOPD. Immunohistochemical staining of NE (a) and MCP-1 (b) proteins in lung tissues (magnification ×100 and ×400). Estimate of NE (c) and MCP-1 (d) expressions through Western blot. *β*-actin was used as an internal control. The values were shown as proportions of target proteins to *β*-actin optical density. Changes in relative mRNA levels of NE (e) and MCP-1 (f) in lung tissues. The values were expressed as mean ± SD. One-way ANOVA was adopted for statistical analysis. ^*∗*^Compared to Control group* P* < 0.05, ^*∗∗*^*P* < 0.01; ^#^compared to AECOPD group* P* < 0.05, ^##^*P* < 0.01; ^&&^compared to CAM group* P* < 0.01.

To observe the infiltration of inflammatory cells in each group, the levels of NE and MCP-1 in rat lung tissues were evaluated by IHC, qPCR, and Western blot analysis. Immunohistochemical staining showed that NE and MCP-1 protein expressions evidently increased in AECOPD group compared to Control group. It could be found that both CAM and LQZS could inhibit the NE and MCP-1 production (Figures 7(a) and 7(b)). Western blot images indicated that stimulation with smoke and LPS could notably increase NE and MCP-1 proteins expressions compared to Control group. After CAM and LQZS treatment NE expression was markedly decreased, and the effect of CAM was more potent (Figure 7(c)). But CAM and LQZS are ineffective in MCP-1 (Figure 7(d)). As for the qPCR results, NE mRNA levels in four groups remained the same (Figure 7(e)). MCP-1 mRNA levels in lung tissues were stable in comparison with normal rats, while treatment with CAM and LQZS evidently decreased the levels (Figure 7(f)).

## 4. Discussion

Exacerbations of COPD, acute worsening of respiratory symptoms, are important events in the management of COPD, because repeated exacerbations impair survival period of COPD patients [1]. Mucus hypersecretion and inflammatory response are the critical pathological factors for AECOPD. Recent studies showed that there are no effective therapies for excess mucus products in the airway. According to TCM theory, the main syndrome of AECOPD is characterized by phlegm-heat obstructing lung. LQZS can clear heat and resolve phlegm. Based on our previous study in clinical, we found that LQZS decoction could improve AECOPD patients' symptoms especially sputum [23]. But the mechanism remains unclear. We designed this research to explore the mechanism of LQZS against mucus hypersecretion. In this study, we successfully established a rat AECOPD model by intratracheal instillation of LPS in combination with the continuous cigarette smoking. Results from lung function showed that parameters like FEV_0.3_/FVC and MVV from AECOPD model group declined. And morphology demonstrated lung injury, inflammatory cells infiltration, and goblet cells hyperplasia in lung tissues of AECOPD model. After treatment with LQZS or CAM, we found that the therapy could significantly improve the MVV, reduce histopathological injury, and inhibit MUC5AC mRNA transcription and protein synthesis in lung tissues of COPD.

MUC5AC is main component of mucus in airway. In recent decades, EGFR-PI3K-AKT signaling pathway has been suggested as an essential role in regulating MUC5AC expression. In the surface epithelium of COPD patients, phosphorylation of EGFR was induced by cigarette smoke and cytokines like EGF, TGF-*α*, VEGF, and TNF-*α* [25]. Activated EGFR caused excess mucin synthesis via triggering a series of intracellular signals ERK1/2 and PI3K/AKT [26]. Western blot images manifested the significant rising in the ratio of phosphorylation of EGFR-PI3K-AKT signaling pathway in COPD lung tissues and a marked suppression after treatment. The results indicated that LQZS and CAM effectively reduced the activation and phosphorylation of EGFR-dependent signaling pathway which contributed to mitigation of airway mucus hypersecretion.

Recently, many evidences indicated that CD4+ T lymphocytes played critical roles in the progression and development of COPD [21, 27]. Th17 cell, a major subset of effector CD4+ T cell, is critically involved in inflammatory and autoimmune responses [28]. T helper (Th) 17 cells and Th17-related cytokines (e.g., IL-17A, IL-6, and IL-23) were reported increases in peripheral blood and lung tissues of clinical patients and laboratory animals with COPD [21, 27, 29]. Th17 activated to differentiate by IL-6, IL-1, and TNF-*α* generates signature cytokine IL-17A. IL-17 is a typical proinflammatory cytokine which can not only recruit inflammatory cells and induce infiltration, but also promote the expression of IL-6, IL-1 and TNF-*α* in turn. ROR*γ*t, the specific transcription factor of Th17, promotes Th17 differentiation and facilities the expression of genes encoding IL-17 [30, 31]. The level of ROR*γ*t was elevated by proinflammatory cytokine via phosphorylation of STAT3 [32]. In this study, we found that IL-6 and IL-17A demonstrated significant increases in BALF of AECOPD rats compared with Control group, whereas the cytokines decreased after administration of CAM and LQZS. The similar change tendency was shown in terms of ROR*γ*t protein level.

CD4+ CD25+ Foxp3+ regulatory T cell (Treg), another subtype of CD4+ T cell, is essential for preventing autoimmunity and maintaining lymphocyte homeostasis by contacting or releasing inhibitory cytokine like IL-10 and TGF-*β* on other immune cells during chronic inflammatory disease [33]. IL-10, primarily produced by Tregs, inhibits the secretion of proinflammatory cytokines and chemokines, and suppresses the differentiation and function of Th17. Meanwhile, IL-10 is involved in improving the differentiation and effect of Tregs [34]. Another anti-inflammatory cytokine TGF-*β* induces Tregs, but depends on local cytokines milieu [35]. TGF-*β* in combination with IL-6 was reported to enhance Th17 generation and suppress Tregs. IL-10 was reported decrease in peripheral blood and BALF in rats with long-time smoke inhalation, whereas the trend was reversed in terms of TGF-*β* [36]. The expression of Foxp3, a specific transcription factor essential for Treg differentiation, is upregulated by IL-10 and TGF-*β* and suppressed by ROR*γ*t. Recent reports demonstrated that Foxp3 deficiency and deletion cause the failure in Tregs generation, resulting in severe autoimmune and inflammatory diseases. It has been shown that the generation and function of Tregs and Treg-related cytokines were reduced in both COPD patients and animals [37]. High Th17/Treg ratio mediated by ROR*γ*t/Foxp3 played a pivotal role in the chronic inflammation of COPD [36]. In this study, the data demonstrated that IL-10 mRNA in lung, as well as Foxp3 protein decreased markedly from rats with AECOPD compared with normal rats and notable increased after LQZS treatment, whereas the result was opposite in TGF-*β* in BALF. The trend was also shown in Foxp3 of CAM group, but the effect of LQZS was more potent. Our research suggested that LQZS might inhibit inflammatory response via regulating Th17/Treg balance.

After MUC5AC synthesis occurred, mucins were tightly packed into granule in cytoplasm of goblet cells and held constant until trigger by extracellular stimuli. One of the most potent stimuli is neutrophil elastase (NE) which is secreted by neutrophil [15]. Th17 cells and cytokines produced by Th17 lead to recruitment and infiltration of neutrophil, monocyte, and macrophage. Activated neutrophils closely contact goblet cells and release NE, which improves mucins exocytosis with the help of adhesion molecules. Besides, NE also stimulates mucin synthesis by EGFR cascade [38]. MCP-1 mainly expressed by monocyte and macrophage could induce mucin production in airway epithelium as well [12]. In this study, the results displayed an obvious increase in NE and MCP-1 proteins of COPD rats compared with Control group, and a decrease in NE of both LQZS and CAM groups, while the effect of CAM was more outstanding.

Recent studies indicated that long-term macrolides therapy could reduce the risk of COPD exacerbations compared to usual care [39]. The mechanism may account for their antibacterial activity, anti-inflammation effect, and immunoloregulation [40]. Researches showed that macrolides consistently reduced MUC5AC synthesis in airway in both vivo and vitro [41, 42]. Although azithromycin was the main macrolide recommended in Global Initiative for Chronic Obstructive 2018 Report, it was associated with increased incidence of bacterial resistance, impaired hearing test and cardiovascular death [43, 44]. It had been proved that clarithromycin (CAM) inhibited proinflammatory cytokines expression (including IL-6, TNF-*α*, and MCP-1) [45], reduced neutrophil infiltration [46] and modulates Th17 response [47] with low adverse event rates [48]. In this study, we found that CAM could decrease the goblet cell hyperplasia, suppress EGFR-PI3K-AKT signaling pathway, and inhibit inflammation via Th17/Treg rebalance. However, the effects were less potent than LQZS.

According to TCM theory, the main syndrome type of AECOPD is “phlegm-heat obstructing lung”. In addition, the imbalance of Th17/Treg in AECOPD associated with Yin and Yang theory [49]. LQZS, a formula designed based on the TCM theory and clinical experience, is consist of eight Chinese herbs. Among these herbs, Gualoupi, Zhebeimu, Loulu, Huangqin, Lianqiao and Qingbanxia can clear lung-heat and remove phlegm, while Qianhu and Jiegeng can regulate lung-qi. Our previous study showed that LQZS effectively relieved sputum and alleviated clinical symptoms of AECOPD patients. It is important to clarify the effect and mechanism of LQZS in mucus hypersecretion of AECOPD. In this study, it could be observed that LQZS significantly alleviated goblet cell hyperplasia and mucus hypersecretion. In further investigation of mechanisms, we found that LQZS had effect on inhibiting phosphorylation of EGFR-PI3K-AKT signaling pathway. Compared with CAM, LQZS was more potent in restoring Th17/Treg balance indicating the effect in maintaining equilibrium between Yin and Yang and thereby protected lung tissues from inflammatory injuries and suppressed mucus release in turn. Combined with our previous clinical study, we recommend AECOPD patients with large amount of yellow sputum, cough, dyspnea, and wheezing taking LQZS decoction to alleviate symptoms. However, LQZS could not replace respiratory support and the medication commonly used for COPD exacerbations such as bronchodilators, corticosteroids, and antibiotics.

## 5. Conclusions

In summary, this study demonstrated that LQZS could inhibit mucus hypersecretion in airway of AECOPD. The mechanism may be associated with downregulation of EGFR-PI3K-AKT signaling pathway. Meanwhile, LQZS could attenuate inflammatory response of lung tissue by restoring Th17/Treg balance, which is also related to mucus release. LQZS can be a therapeutic method of COPD in clinical application, while more mechanism remains to be explored further.

## Figures and Tables

**Table 1:** Primers sequence used for RT-qPCR.

Gene	Primer	Sequence (5′ → 3′)
GAPDH	FW	GCGAGATCCCGCTAACATCA
	RV	CTCGTGGTTCACACCCATCA
MUC5AC	FW	TCATCACTATCTGCGACTAT
	RV	TGATTCTCCAACGCTGTC
ROR*γ*t	FW	TCTGGAAGCTGTGGGATAGA
	RV	GAGGAGCCTGTGGAGAAATAC
Foxp3	FW	GGCCCTTCTCCAGGACAGA
	RV	GCTGATCATGGCTGGGTTGT
IL-17	FW	TGGTCCTGAAGAGGGAGCCT
	RV	TAGGACGCATGGCGGACAAT
IL-10	FW	GCTATGTTGCCTGCTCTT
	RV	CCAAGTAACCCTTAAAGTCC
NE	FW	GGCATCATCTTCATTGTCCTTG
	RV	AGCATTGTCCTCCCACTCG
MCP-1	FW	CATCAACCCTAAGGACTTCAGC
	RV	TCTACAGAAGTGCTTGAGGTGGT

Note. FW: forward; RV: reverse.

**Table 2:** Comparison of pulmonary function parameters in rats (n=6, $\overset{-}{x}\pm s$).

Group	FEV_0.3_ (mL)	FVC (mL)	FEV_0.3_/FVC (%)	MVV (mL/s)
Control	8.77 ± 0.21	13.54 ± 0.46	64.81 ± 2.89	241.25 ± 4.57
AECOPD	7.64 ± 0.36^*∗∗*^	12.93 ± 0.98	59.25 ± 2.94^*∗∗*^	227.56 ± 4.85^*∗∗*^
CAM	7.84 ± 0.31^*∗∗*^	13.06 ± 0.50	60.13 ± 3.30^*∗∗*^	236.69 ± 1.73^##^
LQZS	7.93 ± 0.21^*∗∗*^	13.41 ± 0.31	58.97 ± 1.23^*∗∗*^	242.76 ± 3.84^##&^

Note. The values were expressed as mean ± SD. One-way ANOVA was adopted for statistical analysis. ^*∗∗*^Compared to Control group *P* < 0.01; ^##^compared to AECOPD group *P* < 0.01; ^&^compared to CAM group *P* < 0.05.

## Data Availability

The images of this study are included within the article. The datasets used to support the findings of this study are included within the supplementary materials. More details are available from the corresponding author upon request.

## Supplementary Materials

Supplementary Materials Lung Function.xlsx contains the result of FEV0.3, FVC, FEV0.3/FVC, and MVV. ABPAS.xlsx contains the result of ABPAS-positive staining areas of epithelium. ELISA.xlsx contains the result of IL-6, IL-17, IL-10, TNF-*α*, and TGF-*β*. Western Blot.xlsx contains the result of phosphorylation levels of EGFR, PI3K, and AKT and the level of MUC5AC, NE, MCP-1, ROR*γ*t, and Foxp3. RT-PCR.xlsx contains the result of relative mRNA levels of MUC5AC, NE, MCP-1, ROR*γ*t, Foxp3, IL-17, and IL-10.
